# A Theory

**Published:** 1890-07

**Authors:** W. I. Thayer

**Affiliations:** Brooklyn, N. Y.


### A THEORY.

BY PROF. W. I. THAYER, BROOKLYN. N. Y.

There is not much chance for speculation in infant feeding, when the child is suckled in the natural way ; but artificial infant feeding has been theorized upon by many who have made the treatment of children's diseases a specialty.

It has been claimed by those who have made infant feeding a special study, that cow's milk, *per se*, was not a good substitute for mother's milk, while such claims have been considered by many careless observers as altogether theoretical.

Facts do not support those who form hasty conclusions.

Cow's milk as such is not a good substitute for the maternal breast. Nor is barley and milk, or the addition of any of the cereals for *very young infants*, unless the cereals have been converted into dextrine, and a conversion of certain parts of the cow's milk. Such authorities as Profs. Vaughan, Earl, W. B. Atkinson, Drs. Eustice Smith, Rohé, Forchheimer and others hold to these facts as important considerations in artificial infant feeding.

Cow's milk differs from mother's milk in these essential particulars, in proteins, fat, sugar, inorganic substances and in its *casein.*

This latter substance is the chief element of discord. The casein of cow's milk coagulates into a more or less *indigestible mass,* that few infant's digestive apparatus can reduce, much less get ready for absorbition, and it is here the chief difficulty resides.

On the contrary, the casein in the maternal fluid curdles into a flocculent, *easily divisible* mass, that will not rasp its way through the intestinal tract, beca se it has been reduced and prepared for absorbition, long before it could by any means reach the rectum.

The question now arises, where an infant cannot be fed naturally, can it be furnished with a pabulum that it can digest, and digesting, have *every* one of its growing tissues, fully supplied with necessary building material ? We answer, yes !

With certain manipulations, cow's milk for the artificial feeding of infants is the best substitute for breast milk. These manipulations and changes consist in converting its tough casein into such a condition that, when it is coagulated in the stomach, it will curdle in light flaky masses, that can be easily disintegrated in the stomach and duodenum, just as the casein of human milk is.

This is done by partly pre-digesting the milk with *freshly* prepared pancreatine which has been found, over and over again, to so disintegrate this *leathery protein substance*—which is absolutely necessary for full nutrition—that it can be easily disposed of by the feeble digestive powers of such young beings.

"*Freshly prepared*" pancreatine cannot be found in the shops, so that to expect to obtain reasonable results from what can be obtained, and from inexperienced domestic manipulators, is out of the question. It becomes a necessity then, that a reliable infant food should become an article of commerce, made by some reliable and honest manufacturer. This can be, and is done according to the requirements we have claimed were necessary, and the results from the use of such a prepared artificial food are such as were expected, as the following case in practice will fully illustrate:

Katie F., a five weeks' old child, nursed by her mother, poorly nourished, although her mother was a strong and we woman, 37 years old.

The child was fast and rapidly declining from exhaustion from want of food. Its weight was but ten pounds; features drawn and pinched; *emaciation excessive;* malar eminences, and articulations all over the body, were very prominent.

The attending physician had ordered in addition to the maternal nursing, two tablespoonfuls of cow's milk—*undigested*—two tablespoonfuls of hot water and fifteen drops of bovine. Child could not digest the milk-casein and was constantly rejecting the same by acid raisings. Bowels were so constipated as to have no action for two to three days, and then the fæces had to be removed artificially—a severe case of inanition even in a naturally nursed baby. The child did not need aperients, however mild. It needed to be fed upon some kind of food that it could digest and assimilate, and when that was done the whole problem was solved in two days. We ordered lacto-preparata, first in two, then in three tablespoonful feedings.

The first ingestion of this rational food was retained, and without any interference of the bowels they adjusted themselves the next day, and became and continued regular.

It will be remembered that the mother nursed her child to the best of her ability, and was advised to so continue, yet the child was excessively irritable from constipated bowels and cravings of hunger until fed upon lacto-preparata.

Protein substances, the albuminoids, are very important constituents of an infant food. Without them, no proper and full nutrition can be obtained. This was well exemplified in the above case, where the infant received the protein substances, but could not by any means digest them—mostly the tough casein—which was equivalent to withholding the albumen, fibrin and casein; therefore, there is no wonder that we had a *severe case of inanition* to contend with, which was soon remedied when the protein substances were exhibited so that it *was possible* to digest them. Then there was no difficulty about their appropriation by the starving tissues.

An infant food constructed out of dessicated cow's milk will contain a good proportion of the calcium phosphates which are absolutely necessary to furnish building material for the dental tissues and bones, but more especially for the teeth; a requirement that has been almost wholly overlooked.

There are many important points that are necessary to be observed in artificial infant feeding, and in no one substance can so many valuable necessities be found as are resident in partly predigested cow's milk, as prepared by an honest, intelligent manufacturer, who understands the necessities of infantile digestion and the chemistry of milk.

The raw starches, in an infant food, are very objectionable, from the fact that no infant, under one year old, possesses enough of the amyolytic ferments, in the intestinal tract, to digest such pabulum. If the cereals are roasted at a temperature of 350° F.—which converts them into *dextrine*—the case is very different, for dextrine can be digested quite well in children of six months and upward.

